# COVID-19 Death in High-Medicaid Nursing Homes: The Role of Employment Empowerment

**DOI:** 10.1093/geroni/igab046.2009

**Published:** 2021-12-17

**Authors:** Justin Lord, Ganisher Davlyatov, Akbar Ghiasi, Robert Weech-Maldonado

**Affiliations:** 1 Louisiana State University at Shreveport, Bossier City, Louisiana, United States; 2 University of Alabama at Birmingham, Birmingham, Alabama, United States; 3 University of the Incarnate Word, San Antonio, Texas, United States

## Abstract

This study examines the association between COVID-19 death and employee empowerment in under-resourced nursing homes (70% or higher Medicaid census). Employee empowerment captures elements of participative decision making, autonomy, responsibility, open communication, decentralization, and decision-making flexibility within an organization. Survey data from 391 Directors of Nursing (response rate of 37%) from 2017-2018, were merged with secondary data from CMS Nursing Home COVID-19 Public File, LTCFocus, Area Health Resource File, and Nursing Home Compare. A Poisson regression was used to examine reported COVID-19 death and employee empowerment. The independent variable employee empowerment was the mean score of summated Likert scale questions. Control variables included organizational (size, location, ownership, chain affiliation, quality, payer mix, acuity, occupancy, and race/ethnicity, staffing mix), and county factors (Medicare Advantage penetration, per capita income, poverty, unemployment, education, 65+ population, and competition). Employee empowerment was associated with a lower number of COVID-19 cases (p < 0.05). Rural, not-for-profit, and payer-mix were also significantly associated with a lower number of COVID-19 deaths. Employee empowerment captures the decentralization of authority and an employee’s ability to make decisions without approval. In light of this crisis, empowerment may have helped under-resourced nursing homes be more agile and faster in their response. High-Medicaid nursing homes may need to consider different decision-making practices when faced with a crisis, such as, COVID-19.

